# Risk of breast cancer among Norwegian women with visual impairment

**DOI:** 10.1054/bjoc.2000.1617

**Published:** 2001-02

**Authors:** J Kliukiene, T Tynes, A Andersen

**Affiliations:** The Cancer Registry of Norway, Montebello N-0310, Oslo, Norway; Norwegian Radiation Protection Authority, Kirkevn. 166, Oslo, N-0407, Norway

**Keywords:** blindness, visual impairment, breast cancer, melatonin

## Abstract

Experimental studies suggest that melatonin has a protective effect against breast cancer. Exposure to light suppresses melatonin secretion, but to a lesser degree in totally blind persons. Breast cancer was investigated in a cohort of 15 412 Norwegian visually impaired women. The risk among totally blind women was 0.64 (95% CI = 0.21–1.49, 5 cases only), and for those who became blind before age of 65, the SIR was 0.51 (95% CI = 0.11–1.49). Our findings give support to the ‘melatonin hypothesis’. © 2001 Cancer Research Campaign http://www.bjcancer.com


British Journal of Cancer (2001) 84(3), 397–399
© 2001 Cancer Research Campaign
doi: 10.1054/ bjoc.2000.1617, available online at http://www.idealibrary.com on IDEAL®

### Risk of breast cancer among Norwegian women with visual impairment

**J Kliukiene[1], T Tynes[1,2] and A Andersen[1]**

[1] The Cancer Registry of Norway, Montebello N-0310 Oslo, Norway; [2] Norwegian Radiation Protection Authority, Kirkevn. 166, N-0407 Oslo, Norway

**Summary** Experimental studies suggest that melatonin has a protective effect against breast cancer. Exposure to light suppresses melatonin secretion, but to a lesser degree in totally blind persons. Breast cancer was investigated in a cohort of 15 412 Norwegian visually impaired women. The risk among totally blind women was 0.64 (95% CI = 0.21–1.49, 5 cases only), and for those who became blind before age of 65, the SIR was 0.51 (95% CI = 0.11–1.49). Our findings give support to the 'melatonin hypothesis'. © 2001 Cancer Research Campaign http://www.bjcancer.com

**Keywords**: blindness; visual impairment; breast cancer; melatonin

Breast cancer is a hormone-related disease, and there is ample evidence that ovarian hormones, i.e. oestrogen and progesterone, are of principal importance in its development, especially in post-menopausal women (Key and Pike, 1988; Bernstein and Ross, 1993). Well-established risk factors of the disease such as low parity (Kvåle et al, 1987), high age at first birth (MacMahon et al, 1970), late menopause (Trichopoulos et al, 1972), and early menarche (Henderson et al, 1988) are believed to be associated with increase in oestrogen levels.

In the early 1980s, animal studies indicated that the pineal hormone melatonin inhibits the growth of melanoma and breast cancer in vivo and in vitro (Tamarkin et al, 1981; Narito and Kudo, 1985). In 1987, Stevens proposed the 'melatonin hypothesis' (Stevens, 1987), suggesting that exposure to electric power, including visible light, may reduce pineal production of melatonin and thereby increase breast cancer rates. Specifically, exposure of the eyes to light during the night can cause a rapid decrease in the high nocturnal synthesis and secretion of melatonin. Thus, increased exposure to nocturnal or diurnal visible light may partly be related with increasing incidence of breast cancer (Stevens and Davis, 1996). In totally blind people without ocular perception of light melatonin production is not suppressed by exposure to visible light, therefore a reduced risk of breast cancer could be anticipated. Studies from Sweden and Finland have shown a lower breast cancer risk in totally blind women compared to that of the general female population (Feychting et al, 1998; Verkasalo et al, 1999). A US case-control study also indicated that, overall, women with bilateral blindness had almost half the risk of developing breast cancer compared to the control group (Hahn, 1991).

The aim of our study was to investigate the risk of breast cancer in a Norwegian cohort of blind or milder visually impaired women.

*Received 20 June 2000*
*Revised 7 November 2000*
*Accepted 24 October 2000*

*Correspondence to:* J Kliukiene

### MATERIALS AND METHODS

The present study was based on data from the Norwegian Registry of Blindness, including 15 736 women (Hansen et al, 1996). Medical registration of blind or visually impaired people was initiated in Norway in 1946, and in 1968 the registration became compulsory for all ophthalmologists. Excluded from the study were women who died or emigrated before 01.01.1961 or had no date of registration to the registry (324 persons). For each woman, we had information on date of birth, personal identification number, category of visual impairment, and date of registration of the visual impairment. Data on marital status and age at first birth was obtained from Statistics Norway in 1997. Since age at first birth was only available for women born 1935 or later, this information was of limited value for our study.

The categories of visual impairment were defined by an ophthalmologist according to the classification of the World Health Organisation (WHO 1980): 1 – moderate low vision (visual acuity less than 0.3); 2 – severe low vision (visual acuity less than 0.12, i.e., able to count fingers at 5 m); 3 – profound low vision or moderate blindness (visual acuity less than 0.05, i.e., finger counting at <3 m, or finger counting NOS); 4 – near total blindness (visual acuity less than 0.02, i.e., finger counting at <1 m, or light perception); and 5 – total blindness (no light perception). The latest degree of the impairment with best correction was used in the analysis.

Tables 1 and 2 provide the distribution of the material by degree of visual impairment, by age at onset of total blindness or registration of latest visual impairment, and by marital status. In the total cohort, 3% of the subjects were totally blind. Among the totally blind women, 69% became blind before age of 65, and 47% were single, while the visual impairment of the majority of women with milder degree of visual impairment (74%) was registered at age 65 or higher, and only 20% of these women were never married.

The follow-up period was from January 1, 1961 to the end of 1997. The start of the follow up was the date of blindness, if any, or the date of registration of the latest visual impairment. January 1, 1961 was defined as the start of follow up for women who became blind or were registered before the beginning of the

**Table 1** Number of Norwegian women with visual impairment and person-years at risk in the period 1961–1997 by degree of visual impairment

| Degree of visual Impairment | Number of women | | Person-years | |
|---|---|---|---|---|
| | *n* | % | *n* | % |
| Moderate low vision | 6104 | 40 | 53150 | 35 |
| Severe low vision | 3153 | 20 | 29486 | 20 |
| Profound low vision | 2987 | 19 | 28840 | 19 |
| Near-total blindness | 2270 | 15 | 26247 | 18 |
| Total blindness | 396 | 3 | 6147 | 4 |
| Unknown | 502 | 3 | 6560 | 4 |
| Total | 15412 | 100 | 150430 | 100 |

overall follow up period. Person-years at risk were calculated from the year of entering the study to the date of death or emigration, or to the end of follow up, December 31, 1997, whichever came first (150 430 person-years). All members of the cohort were linked by their personal identification number to the national registration of date of death or emigration and to the Cancer Registry of Norway where all types of cancer were identified.

The analysis was based on a computation of observed and expected cancer cases. The incidence of cancer in the total female population of Norway served as a reference entity. The expected numbers of cancer cases were calculated by using the 5-year, age-specific incidence rates for the reference entity for each year from 1961 to 1997. Standardized incidence ratios (SIRs) were calculated and 95% confidence intervals (95% CI) were determined by assuming a Poisson distribution of the observed cases with the use of a two-sided test of significance. The actual computation was performed using a standard computer program (Preston et al, 1993).

### RESULTS

The present study included 308 breast cancer cases and 1347 cancers of other sites. The overall SIR was 1.02 (95% CI = 0.91–1.14) for breast cancer and 1.04 (95% CI = 0.99–1.10) for all other cancers combined. The SIRs differed by degree of visual impairment (Table 3). In totally blind women the SIR for breast cancer was 0.64 (95% CI = 0.21–1.49), while for women with milder degree of visual impairment the SIRs varied from 0.92 to 1.22. The SIR for breast cancer was 0.51 based on 3 cases (95% CI = 0.11–1.49) in women who became totally blind before age of 65, while the SIR was 0.02 (2 cases, 95% CI = 0.12–3.69) for those who first experienced blindness at age 65 or later. The same pattern was not observed in women with milder degree of visual impairment (not in table). Among ever married totally blind women we observed two breast cancer cases (SIR = 0.42, 95% CI = 0.05–1.54) and 3 cases for never married totally blind women (SIR = 0.97, 95% CI = 0.20–2.85, not in table).

For other types of cancer combined, the SIRs for all categories were similar to the general female population, varied from 1.00 to 1.08.

**Table 2** Number of Norwegian women with visual impairment and person-years at risk in the period 1961–1997 by age at onset of blindness or visual impairment and by marital status

| | Totally blind | | | | Visually impaired but not blind | | | |
|---|---|---|---|---|---|---|---|---|
| | Number of women | | Person-years | | Number of women | | Person-years | |
| | *n* | % | *n* | % | *n* | % | *n* | % |
| Age at onset of blindness or milder visual impairment (years) | | | | | | | | |
| <15 | 126 | 32 | 2939.6 | 48 | 821 | 6 | 18816.9 | 14 |
| 15–64 | 148 | 37 | 2427.8 | 39 | 2861 | 20 | 43712.6 | 32 |
| 65+ | 122 | 31 | 779.9 | 13 | 10832 | 74 | 75193.0 | 54 |
| Total | 396 | 100 | 6147.3 | 100 | 14514 | 100 | 137723.0 | 100 |
| Marital status | | | | | | | | |
| Ever married | 206 | 52 | 2734.1 | 44 | 11265 | 78 | 101854.0 | 74 |
| Never married | 186 | 47 | 3381.6 | 55 | 2986 | 20 | 34217.0 | 25 |
| Unknown | 4 | 1 | 31.6 | 1 | 263 | 2 | 1651.6 | 1 |
| Total | 396 | 100 | 6147.3 | 100 | 14514 | 100 | 137723.0 | 100 |

**Table 3** Standardized incidence ratios (SIRs) of breast cancer and of all other cancers except breast combined by degree of visual impairment

| Degree of visual impairment | Breast cancer | | | Other cancers, except breast | | |
|---|---|---|---|---|---|---|
| | Obs. | SIR | 95% CI | Obs. | SIR | 95% CI |
| Moderate low vision | 104 | 0.92 | 0.76–1.11 | 534 | 1.08 | 0.99–1.17 |
| Severe low vision | 77 | 1.22 | 0.96–1.52 | 274 | 1.00 | 0.89–1.12 |
| Profound low vision | 63 | 1.00 | 0.77–1.29 | 274 | 1.01 | 0.90–1.14 |
| Near-total blindness | 57 | 1.21 | 0.92–1.57 | 197 | 1.02 | 0.89–1.17 |
| Total blindness | 5 | 0.64 | 0.21–1.49 | 32 | 1.07 | 0.73–1.51 |
| Unknown | 2 | 0.27 | 0.03–0.99 | 36 | 1.30 | 0.91–1.79 |
| Total | 308 | 1.02 | 0.91–1.14 | 1347 | 1.04 | 0.99–1.10 |

*© 2001 Cancer Research Campaign*

### DISCUSSION

The present study showed a low risk of breast cancer among totally blind women, but the finding was based on few cases. Our results are consistent with findings from Sweden (Feychting et al, 1998) and Finland (Verkasalo et al, 1999). However, we did not observe any decrease of breast cancer risk in the Norwegian women with milder degree of visual impairment, and in this respect our results correspond with the Swedish, but differ from the Finnish results. The Finnish authors reported a reduced risk in most of the categories of visual impairment and a decreasing trend by the categories. Our finding of an association before age of 65 at onset of blindness also corresponds with the results from the case-control study in US (Hahn, 1991). In women with milder degree of visual impairment, we did not observe the same difference in the risk by age at onset of visual impairment as we did in totally blind women.

Marital status was used in our study as an indicator of parity. Never married totally blind women had twice the risk of breast cancer compared to ever married blind women. The risk of breast cancer in never married blind women did not appear to be higher than in the total general population, while never married women with a milder degree of visual impairment had a 39% excess risk, which altogether indicate a protective effect of blindness. Nulliparity is a well-established risk factor of breast cancer, and a high proportion of nulliparous women was registered among blind women. In our study, as much as 89% of totally blind women born in 1935 or later were nulliparous, while only 39% of all Norwegian women born in the same period had not given birth.

The low risk observed in totally blind women without light perception is in agreement with the ‘melatonin hypothesis’. Melatonin is a hormone involved in the regulation of the female menstrual cycle and circadian rhythm by altering the firing rate of the gonadotropins and prolactin and, indirectly, the secretion of oestrogen by the gonads (Tamarkin et al, 1985). Current knowledge of melatonin receptors provides theories on how the inactivation of these receptors may cause cancer (Baldwin and Barrett, 1998). The presence of melatonin suppresses cancer growth in experimental animals and in cell cultures (Liburdy et al, 1993; Mevissen et al, 1996). It is known that light suppresses the nocturnal peak of melatonin secretion in humans (Lewy et al, 1980). Since most totally blind women are not (ocularly) receptive to light, they might not have the potential for decreased melatonin production by light at night and could thus be protected from breast cancer through this mechanism (Hahn, 1991). In 1992 Coleman and Reiter suggested that a retrospective cohort study of blind women, carried out by linking a comprehensive registry of blind persons to a long-standing cancer registry of adequate quality, would be able to test the hypothesis that long-term blindness protects against breast cancer (Coleman and Reiter, 1992).

Our study showed a reduced risk of breast cancer among totally blind women where age at onset of blindness was important and our findings give support to the ‘melatonin hypothesis’. Therefore a multicentre study providing a higher number of breast cancer cases would be of interest.

### ACKNOWLEDGEMENTS

This work was financially supported by Telenor AS. The authors express thanks to Tor Flage and Egill Hansen for their contributions to this project. The authors also acknowledge Jan Ivar Martinsen and Lars Klæboe for the assistance in computing and data registration.

### REFERENCES

Baldwin WS and Barrett JC (1998) Melatonin: receptor-mediated events that may affect breast and other steroid hormone-dependent cancers. Review. *Mol Carcinog* **21**(3): 149–155

Bernstein L and Ross RK (1993) Endogenous hormones and breast cancer risk. Review. *Epidemiol Rev* **15**(1): 48–65

Coleman MP and Reiter RJ (1992) Breast cancer, blindness and melatonin. *Eur J Cancer* **28**: 501–503

Feychting M, Osterlund B and Ahlbom A (1998) Reduced cancer incidence among the blind. *Epidemiology* **9**: 490–494

Hahn RA (1991) Profound bilateral blindness and the incidence of breast cancer. *Epidemiology* **2**: 208–210

Hansen E, Falch B and Quale GA (1996) *Det Norske Blindekartotek 1968–1995*. Oslo, (In Norwegian)

Henderson BE, Ross R and Bernstein L (1988) Estrogens as a cause of human cancer: the Richard and Hinda Rosenthal Foundation Award lecture. *Cancer Res* **48**: 246–253

Key TJ and Pike MC (1988) The role of oestrogens and progestagens in the epidemiology and prevention of breast cancer. Review. *Eur J Cancer Clin Oncol* **24**(1): 29–43

Kvåle G, Heuch I and Eide GE (1987) A prospective study of reproductive factors and breast cancer. I. Parity. *Am J Epidemiol* **126**: 831–841

Lewy AJ, Wehr TA, Goodwin FK, Newsome DA and Markey SP (1980) Light suppresses melatonin secretion in humans. *Science* **210**: 1267–1269

Liburdy RP, Sloma TR, Sokolic R and Yaswen P (1993) ELF magnetic fields, breast cancer, and melatonin: 60 Hz fields block melatonin’s oncostatic action on ER+ breast cancer cell proliferation. *J Pineal Res* **14**(2): 89–97

MacMahon B, Lin TM, Lowe CR, Mirra AP, Ravnihar B, Salber EJ, Trichopoulos D, Valaoras VG and Yuasa S (1970) Age at first birth and cancer of the breast. A summary of an international study. *Bull World Health Organ* **43**: 209–221

Mevissen M, Lerchl A, Szamel M and Loscher W (1996) Exposure of DMBA-treated female rats in a 50-Hz, 50 microTesla magnetic field: effects on mammary tumor growth, melatonin levels, and T lymphocyte activation. *Carcinogenesis* **17**(5): 903–910

Narito T and Kudo H (1985) Effect of melatonin on B16 melanoma growth in athymic mice. *Cancer Res* **45**: 4175–4177

Preston DL, Lubin JH, Pierce D and McConney M (1993) *Epicure*. Seattle: HiroSoft International

Stevens RG (1987) Electric power use and breast cancer: a hypothesis. *Am J Epidemiol* **125**: 556–561

Stevens RG and Davis S (1996) The melatonin hypothesis: electric power and breast cancer. *Environ Health Perspect* **104**: 135–140

Tamarkin L, Cohen M and Roselle D (1981) Melafouronin inhibition and pinealectomy enhancement of 7,12-dimethylbenz(a)anthracene-induced mammary tumors in the rat. *Cancer Res* **41**: 4432–4461

Tamarkin L, Baird CJ and Almeida OFX (1985) Melatonin: a coordinating signal for mammalian reproduction? *Science* **227**: 714–720

Trichopoulos D, MacMahon B and Cole P (1972) Menopause and breast cancer risk. *J Natl Cancer Inst* **48**: 605–613

Verkasalo PK, Pukkala E, Stevens RG, Ojamo M and Rudanko SL (1999) Inverse association between breast cancer incidence and degree of visual impairment in Finland. *Br J Cancer* **80**(9): 1459–1460

World Health Organization (1980) *International Classification of Impairments, Disabilities and Handicaps. A Manual of Classification Relating to Consequences of Disease*. Geneva: World Health Organization

*© 2001 Cancer Research Campaign*
